# Report of the Texas peanut root-knot nematode, *Meloidogyne haplanaria* (Tylenchida: Meloidogynidae) from American pitcher plants (*Sarracenia* sp.) in California

**DOI:** 10.21307/jofnem-2021-077

**Published:** 2021-09-02

**Authors:** Sergei A. Subbotin

**Affiliations:** 1 California Department of Food and Agriculture, Plant Pest Diagnostics Center, 3294 Meadowview Road, Sacramento, CA, 95832

**Keywords:** California, *Meloidogyne haplanaria*, Phylogeny, Texas peanut root-knot nematode

## Abstract

During the winter and spring of 2021, the root-knot nematodes were extracted from samples of galled roots of potted American pitcher plants (*Sarracenia* sp.). Samples were collected from a botanical garden nursery in Los Angeles County, California. The root-knot nematode was identified by molecular methods as *Meloidogyne haplanaria*. In the USA, *M. haplanaria* was initially found in Texas, and subsequently reported from Arkansas and Florida. Molecular characterization of the Californian *M. haplanaria* isolate was done using the analysis of the D2-D3 of 28S rRNA, ITS rRNA, mitochondrial l-rRNA, *COI*, and *nad5* gene sequences. Some rRNA gene clusters of *M. haplanaria* were similar with those of *M. arenaria*. Possible hybridization events within mitotic parthenogenetic root-knot nematodes are discussed. This study confirmed that reliable diagnostics of *M. haplanaria* should be based on mtDNA sequence analysis. This is a first report of *M. haplanaria* from *Sarracenia* sp. and California. Consequently, this nematode was considered to be eradicated from this botanical garden nursery and the State of California.

During February and May 2021, several potted American pitcher plants (*Sarracenia* sp.) with roots galls induced by root-knot nematodes were collected from a botanical garden in Los Angeles County, California. Based on the analysis of several molecular markers, the root-knot nematode extracted from the galled roots was identified as the Texas peanut root-knot nematode *M. haplanaria* (Eisenback et al., 2003) in the Nematology Laboratory, Plant Pest Diagnostics Center, California Department of Food and Agriculture, Sacramento, California.

*Meloidogyne haplanaria* was initially described from roots of peanut in Collingsworth, Texas, USA (Eisenback et al., 2003) and its current distribution of *M. haplanaria* includes Texas, Arkansas, and Florida (Joseph et al., 2016). Hosts of *M*. *haplanaria* include tomato, common bean, garden pea, radish, and soybean. This species has also been found in rhizosphere soil of Indian hawthorn, okra, ash, oak, cherry laurel, maple, tomato, willow, rivercane, elm, bermudagrass, and birch in Arkansas (Khanal et al., 2016; Ye et al., 2019). *Meloidogyne haplanaria* has been shown to overcome the *Mi* resistant gene to *M. arenaria, M. javanica*, and *M. incognita* in tomato. However, the peanut cultivar ‘NemaTAM’, which is resistant to *M. arenaria* and *M.javanivca*, is also resistant to the Texas root-knot nematode (Bendezu et al., 2004).

The objective of the present study was to provide molecular characterization of *M. haplanaria* parasitising potted American pitcher plants in a nursery, in California, USA.

## Materials and methods

### Nematode extraction and morphological examination

**Figure 1:**
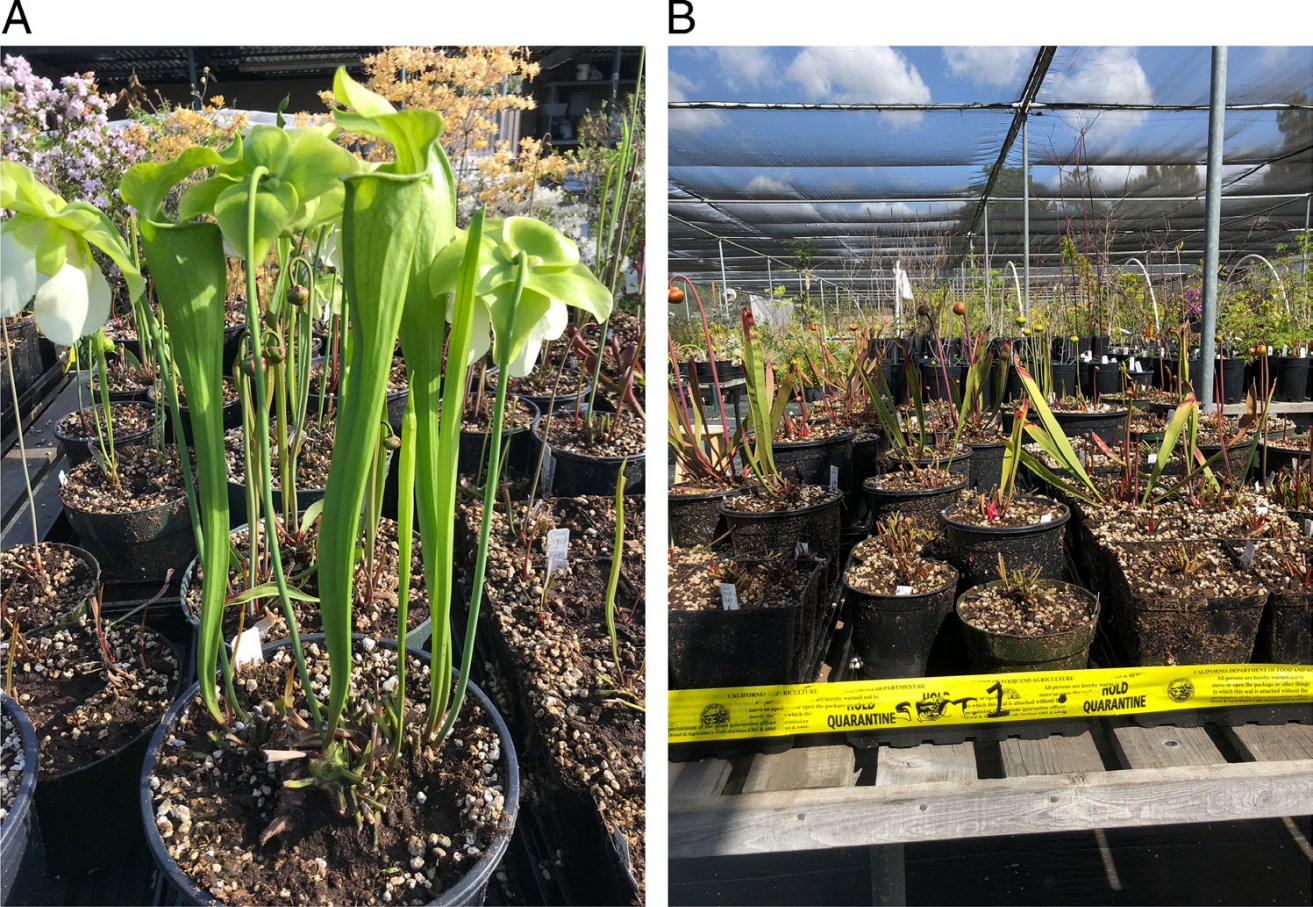
(A) American pitcher plants (*Sarracenia* sp.); (B) area of plant nursery under a quarantine.

Galled roots of American pitcher plants (*Sarracenia* sp.) were collected from potted plants in a botanical garden, Los Angeles County, California (Fig. 1). Nematodes were extracted using the Baermann funnel method. Several second-stage juveniles (J2) and males killed by heating were morphologically examined and microphotographed using an automatic Infinity 2 camera attached to a compound Olympus BX51 microscope equipped with Nomarski interference contrast.

### Molecular analysis of nematode samples

DNA was extracted from several J2 specimens using the proteinase K protocol. DNA extraction and PCR protocols were as described by Janssen et al. (2016) and Subbotin (2021a). The following primer sets were used in this study: D2A (5′-ACA AGT ACC GTG AGG GAA AGT TG-3′) and D3B (5′-TCG GAA GGA ACC AGC TAC TA-3′) amplifying the D2-D3 expansion segments of 28S rRNA gene, TW81 (5′-GTT TCC GTA GGT GAA CCT GC-3′) and AB28 (5′-ATA TGC TTA AGT TCA GCG GGT-3′) amplifying the ITS rRNA gene, NAD5F2 (5′ -TAT TTT TTG TTT GAG ATA TAT TAG-3′) and NAD5R1 (5′ -CGT GAA TCT TGA TTT TCC ATT TTT-3′) amplifying the partial mitochondrial *nad*5 gene, TRANAH (5′ -TGA ATT TTT TAT TGT GAT TAA-3′) and MRH106 (5′-AAT TTC TAA AGA CTT TTC TTA GT-3′) amplifying the partial mitochondrial l-rRNA gene, J3 (5′-TTT TTT GGG CAT CCT GAG GTT TAT-3′) and J4.5 (5′ -TAA AGA AAG AAC ATA ATG AAA ATG-3′) amplifying the partial mitochondrial *COI* gene (Humphreys-Pereira et al., 2021; Subbotin, 2021a). The new sequences for each gene were aligned using ClustalX 1.83 with their corresponding published gene sequences of *M. haplanaria* and other root-knot nematode species (Alvarez-Ortega et al., 2019; Georgi and Abbott, 1998; Joseph et al., 2016; Khanal et al., 2016; Powers et al., 2005; Ye et al., 2019 and others). Sequence datasets were analyzed with Bayesian inference (BI) using MrBayes 3.1.2 as described by Subbotin (2021b). The new sequences were submitted to the GenBank database under accession numbers: MZ050223 ((D2-D3 of 28S rRNA gene), MZ048738 (ITS rRNA gene), MZ048743 (*COI* gene), MZ081011 (l-rRNA gene), MZ081010 (*nad5*) as indicated in the phylogenetic trees.

## Results

### Morphological study

**Figure 2:**
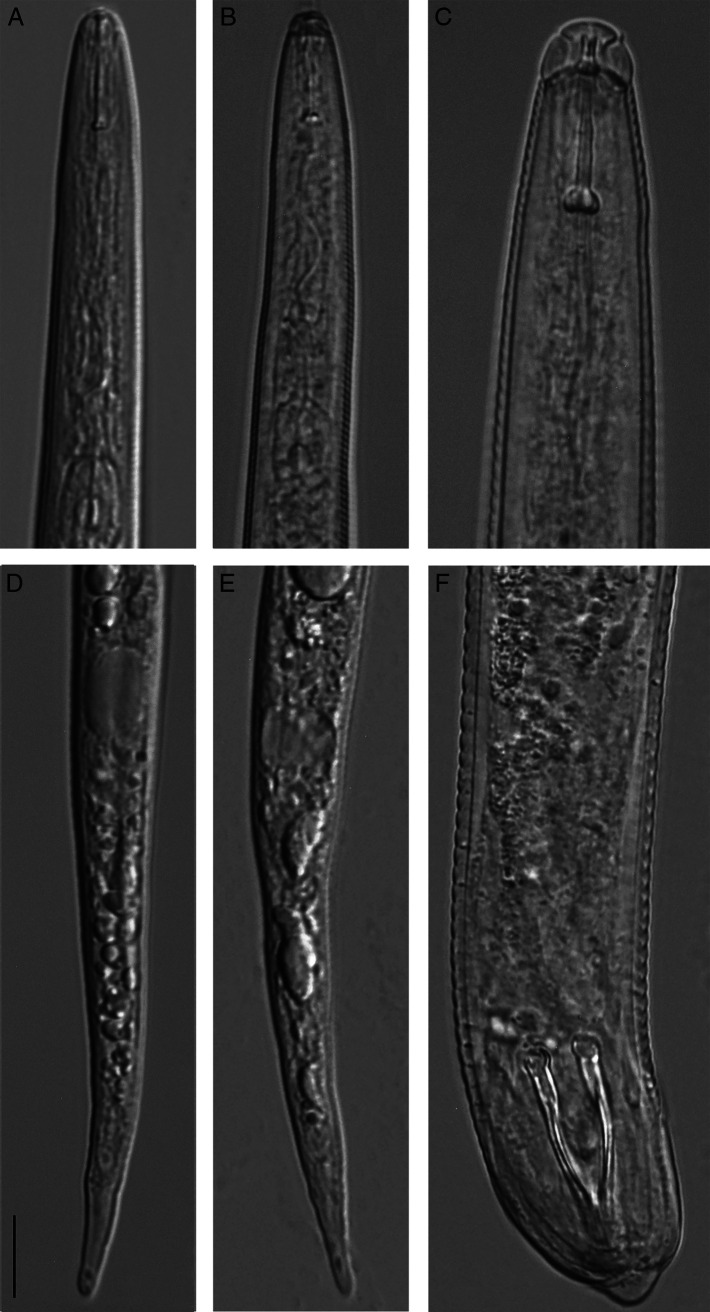
*Meloidogyne haplanaria*. (A, B) Anterior region of J2s; (C) anterior region of male; (D, E) posterior region of J2s; (F) posterior region of male. Scale = 10 μm.

The morphology and morphometrics of J2s and males were similar to those of *Meloidogyne haplanaria* isolates from Texas and Florida (Eisenback et al., 2003; Joseph et al., 2016). J2s from California (*n* = 10): *L* = 447.5 ± 34.0 (362.5–485) µm; stylet length = 13.7 ± 0.8 (12.5–14.6) µm; anterior end to median bulb = 71.0 ± 2.0 (67–72.5) µm; tail length = 67.7 ± 3.6 (62.5–71.3) µm; hyaline region of tail = 15.2 ± 1.5 (12.5–17.5) µm. Body annuli distinct. Labial region high, offset from body. Cephalic framework weakly sclerotised. Stylet delicate with rounded knobs. Anus poorly visible. Tail slender, with slightly round tip (Fig. 2).

### Molecular study

#### The D2-D3 of 28S rRNA gene

**Figure 3:**
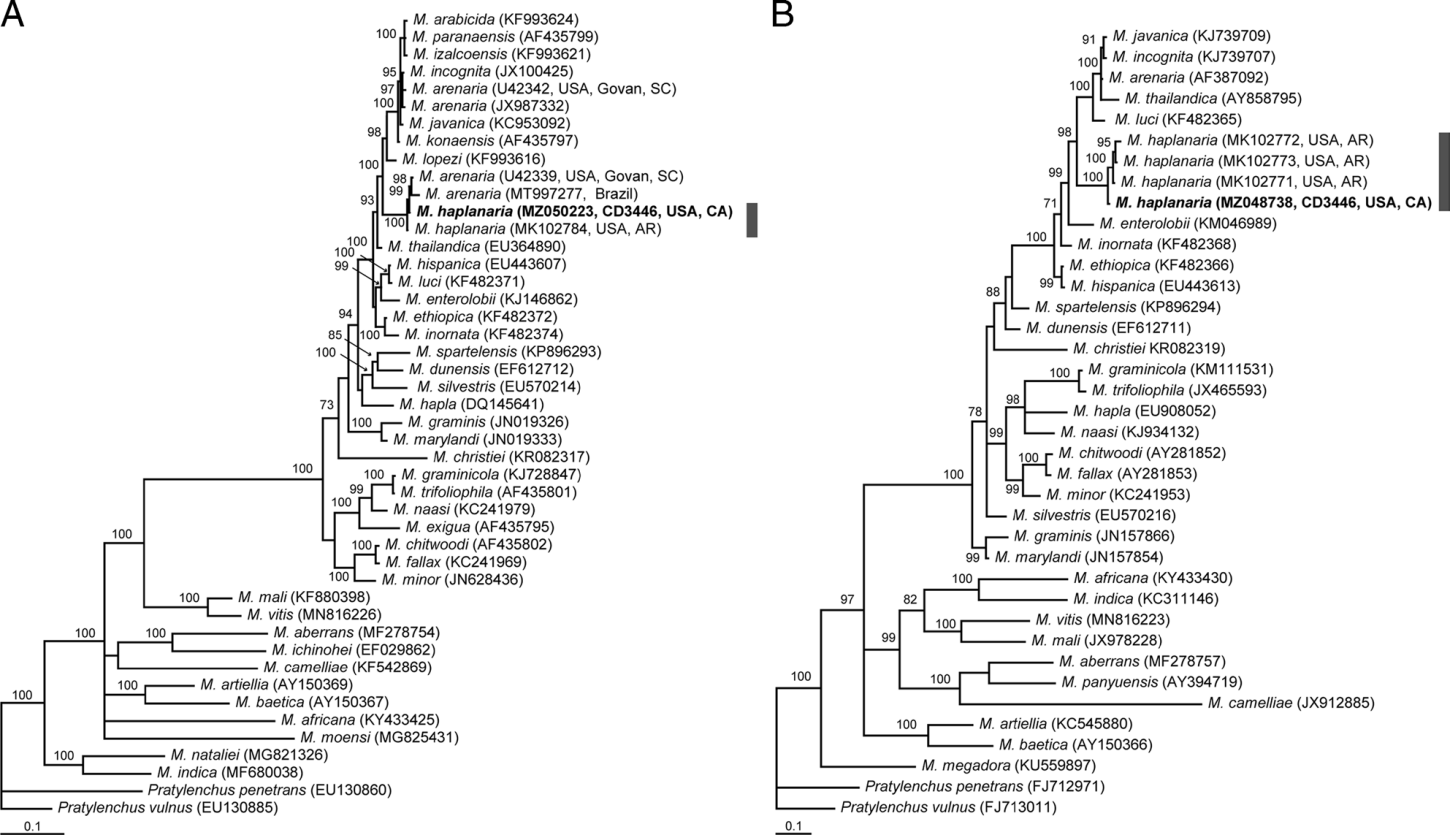
Phylogenetic relationships within *Meloidogyne* spp. Bayesian 50% majority rule consensus trees from two runs as inferred from analyses of the D2-D3 of 28S rRNA (A) and ITS rRNA (B) gene sequence alignments under the GTR + I + G model. Posterior probabilities equal or more than 70% are given for appropriate clades. New sequences are indicated in bold. Grey line indicates *M. haplanaria* sequences.

The alignment was 784 bp in a length and contained 44 sequences of *Meloidogyne* species. Phylogenetic relationships of *M. haplanaria* with other the root-knot nematodes are given in Figure 3A. Sequences of *M. haplanaria* from California and Arkansas formed a clade with two sequences of *M. arenaria* from South Carolina and Brazil. Sequence of *M. haplanaria* from California differed in 1 bp (0.1%) from those of *M. haplanaria* from Arkansas and *M. arenaria* (Govan population, clone BA#4, U42339) from South Carolina and in 35 bp (5.3%) from that of *M. arenaria* (Govan population, clone BA#3, U42342).

#### The ITS of rRNA gene

The alignment was 526 bp in a length and contained 36 sequences of *Meloidogyne* species. Phylogenetic relationships of *M. haplanaria* with other the root-knot nematodes are given in Figure 3B. Sequence of *M. haplanaria* from California formed a clade with those of representatives of this species from Arkansas and differed from them in 3–6 bp (0.8–1.7%).

#### The partial mitochondrial l-rRNA gene

**Figure 4:**
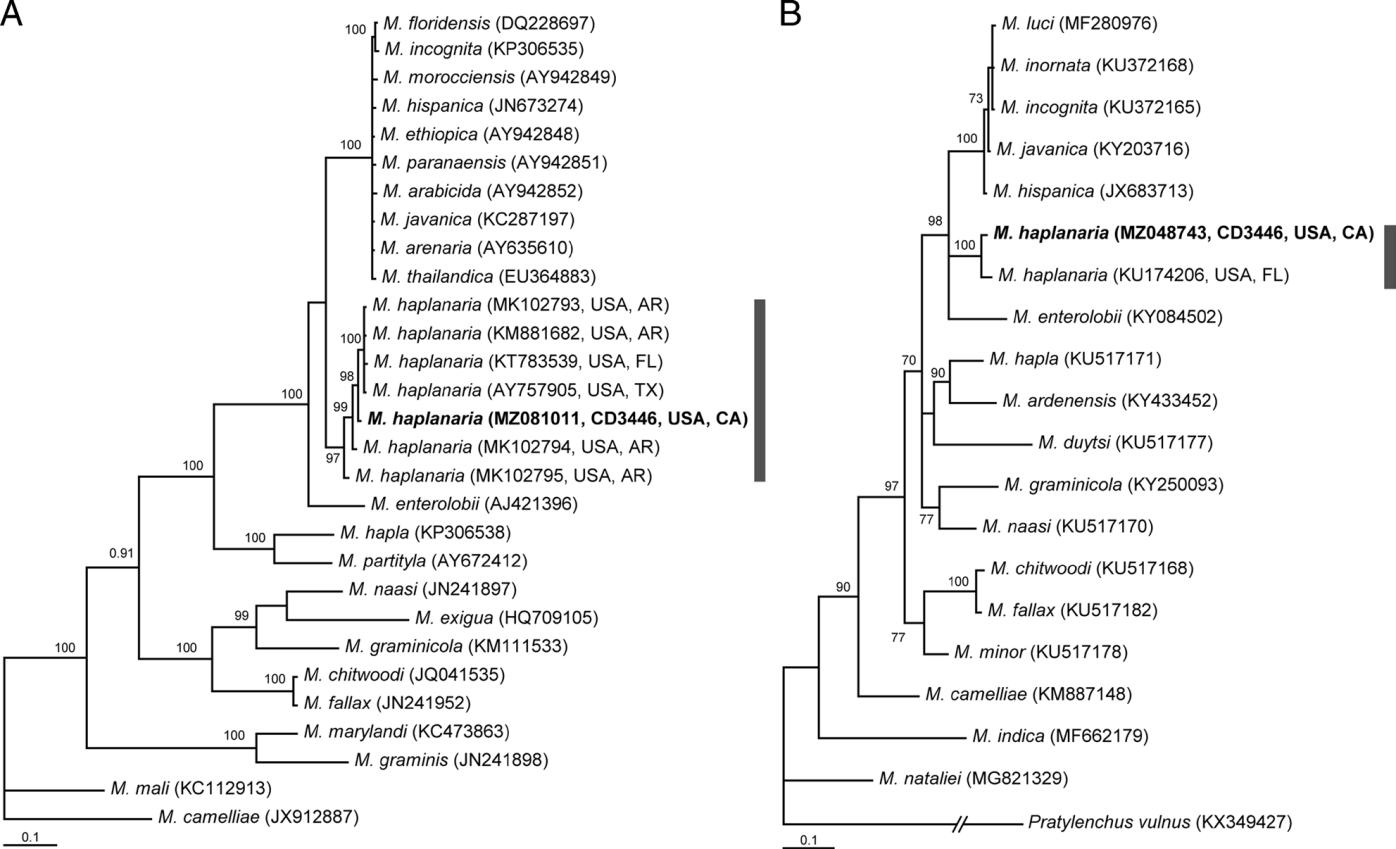
Phylogenetic relationships within *Meloidogyne* spp. Bayesian 50% majority rule consensus trees from two runs as inferred from analyses of the partial large subunit RNA (A) and partial *COI* (B) gene sequence alignments under the GTR + I + G model. Posterior probabilities equal or more than 70% are given for appropriate clades. New sequences are indicated in bold. Grey line indicates *M. haplanaria* sequences.

The alignment was 421 bp in a length and contained 29 sequences of *Meloidogyne* species. Phylogenetic relationships of *M. haplanaria* with other the root-knot nematodes are given in Figure 4A. Sequence of *M. haplanaria* from California clustered with other sequences of this species and differed from them in 4–9 bp (1.0–2.3%).

#### The partial *COI* gene

The alignment was 442 bp in a length and contained 19 sequences of *Meloidogyne* species. Phylogenetic relationships of *M. haplanaria* with other the root-knot nematodes are given in Figure 4B. Sequence of *M. haplanaria* from California clustered with sequence of this species from Florida and differed from it in 9 bp (2.3%).

#### The partial *nad5* gene

Search of *nad5* sequence with Blastn in the Genbank showed 89.2% similarity (100% coverage) with *nad5* sequences of *M. incognita* (MN106036, MN106033, and KJ476151).

## Discussion

The Texas peanut root-knot nematode was molecularly characterized using mtDNA genes (Joseph et al., 2016; Khanal et al., 2016; Powers et al., 2005) and rRNA (Ye et al., 2019). Our study also showed distinct discrimination of *M. haplanaria* from other root-knot nematodes using mitochondrial l-rRNA, *COI* and *nad5* genes. Therefore, mtDNA genes should be considered as more reliable markers for diagnostics of *M. haplanaria* than rRNA genes.

It is noteworthy that the sequences of the D2-D3 of 28S rRNA gene of *M. haplanaria* from California and Arkansas are very similar with those of two *M. arenaria* populations. One of these *M. arenaria* belongs to the Govan population of *M. arenaria*, race 2, which has been well characterized in several publications (Carpenter and Lewis, 1991; Carpenter et al., 1992; Dong et al., 2001; Powers and Harris, 1993). It has been shown that the Govan population was more aggressive on a wider range of hosts than other populations of *M. arenaria* (Carpenter and Lewis, 1991; Hiatt et al., 1988). Georgi and Abbott (1998) reported the presence of two different IGS rRNA gene region copies in individual *M.arenaria* females of the Govan population. One of the sequences (clone BA#3) was identical or similar with those of *M. arenaria*, *M. javanica*, and *M. incognita* published by Blok et al. (1997) and the second sequence (clone BA#4) showed a highest match with the IGS rRNA gene region of *M. enterolobii.* Our analysis showed similar results with the analysis of the D2-D3 expansion segments of 28S rRNA gene sequence and revealed two copies of this gene for the Govan population: one copy clustered with other *Meloidogyne* sequences from the tropical complex, and another copy was similar with those of *M. haplanaria*. Georgi and Abbott (1998) believed that an explanation for this observation was that this *M*. *arenaria* arose by interspecific hybridization, and one (at least) of the parental species was shared with *M. javanica* and *M. incognita*, but descendants of the other parental species have yet to be identified that time. Following this hypothesis and considering our present results, we suggest that the other parental species could be *M. haplanaria*. Our unpublished results with the primer for the D2 of 28S rRNA gene specifically designed for *M. haplanaria* and the universal D3B primer showed amplification with several *M. arenaria* populations indicating that similar *M. haplanaria* rRNA gene copy might be present not only in the Govan population, but also in other *M. arenaria* populations.

It has been shown in several studies that high variation in rRNA gene sequences occurred for some *Meloidogyne* species belonging to the Clade I. For example, the ITS rRNA gene diversity was structured into two groups for *M*. *enterolobii*, *M*. *paranaensis* and some species of the *Ethiopica* group, which ITS rRNA paralogs also clustered within the *Incognita* group (Alvarez-Ortega et al., 2019). Although the hypothesis of hybrid origin of these parthenogenetic species from amphimictic species could be applied to explain this phenomenon, another hypothesis on occurring of hybridization events between modern parthenogenetic *Meloidogyne* species within the Clade I could be considered. However, this hypothesis contradicts with present day knowledge on reproduction modes in *Meloidogyne* species belonging to the Clade I. It has been presently accepted that the root-knot nematodes of the Clade I reproduce exclusively by obligate mitotic parthenogenesis (except for *M. floridensis* with meiotic parthenogenesis). However, males can be sporadically observed in these species and they are able to produce sperm and mate with females, but although sperm can be occasionally observed in the female spermatheca and the sperm nucleus can reach the egg, it has been reported that the sperm nucleus disintegrates in the egg cytoplasm during or just following mitotic division and thus apparently never fuses with the egg nucleus (Castagnone-Sereno et al., 2013; Triantaphyllou, 1981). However, if we can suggest that males may play an active role in reproduction under certain conditions and these nematodes reproduce by facultative mitotic parthenogenesis, observed high variation in rRNA genes can be explained by modern hybridization events between species. It has been known that hybridization between two meiotic parthenogenetic species of root knot nematodes, *Meloidogyne chitwoodi* and *M. fallax* was confirmed by Van der Beek and Karssen (1997). These authors regularly observed hybrids between *M. chitwoodi* and *M. fallax.* Although deformed and nonviable, a very few juveniles were found in the offspring of these hybrids, it was suggested that hybrids might occasionally produce fertile progeny, because they were able to backcross to one of the parents and thus, transmission of the hybrid genome to the next generation cannot excluded. More detailed research on reproduction of mitotic parthenogenetic species should be done to understand reproduction modes and reasons for high intraspecific rRNA gene diversity for these nematodes.

Thus, this is the first report of *M. haplanaria* from a *Sarracenia* plant. The origin of plant materials at the botanical garden nursery remains unknown. However, it cannot be excluded that the *Sarracenia* plants originated from a native habitat. These carnivorous plants with leaves having a funnel or pitcher shape to trap insects is indigenous to the eastern coastal regions of the United States, Texas, the Great Lakes area, and southeastern Canada, with most species occurring in the southeast United States. This was also the first report of *M. haplanaria* in California, however, consequently, all infected potted *Sarracenia* plants from this nursery were destroyed and a comprehensive analysis of composite root and soil samples from plants growing in this area and other places, in which plants might be transferred, did not reveal any specimens of this root-knot nematode species. Thus, the California Department of Food and Agriculture considers the Texas peanut root-knot nematode to be eradicated from this botanical garden nursery and the State of California (J.J. Chitambar, pers. comm.).

## References

[R1] Alvarez-Ortega, S., Brito, J. A. and Subbotin, S. A. 2019. Multigene phylogeny of root-knot nematodes and molecular characterization of *Meloidogyne nataliei* Golden, Rose & Bird, 1981 (Nematoda: Tylenchida). Scientific Report 9:11788. 10.1038/s41598-019-48195-0 PMC6692364 31409860

[R2] Bendezu, I., Morgan, T. and Starr, J. 2004. Hosts for *Meloidogyne haplanaria*. Nematropica 34:205–209.

[R3] Blok, V. C., Phillips, M. S. and Fargette, M. 1997. Comparison of sequence differences in the intergenic region of the ribosomal cistron of *Meloidogyne mayaguensis* and the major tropical root-knot nematodes. Journal of Nematology 29:16–22. 19274129 PMC2619761

[R4] Carpenter, A. S. and Lewis, S. A. 1991. Aggressiveness and reproduction of four *Meloidogyne arenaria* populations on soybean. Journal of Nematology 23:232–238. 19283118 PMC2619152

[R5] Carpenter, A. S., Hiatt, E. E., Lewis, S. A. and Abbott, A. G. 1992. Genomic RFLP analysis of *Meloidogyne arenaria* race 2 populations. Journal of Nematology 24:23–28. 19283197 PMC2619242

[R6] Castagnone-Sereno, P., Danchin, E. G. J., Perfus-Barbeoch, L. and Abad, P. 2013. Diversity and evolution of root-knot nematodes, genus *Meloidogyne*: new insights from the genomic era. Annual Review of Phytopathology 51:203–220. 10.1146/annurev-phyto-082712-102300 23682915

[R7] Dong, K., Dean, R. A., Fortnum, B. A. and Lewis, S. A. 2001. Development of PCR primers to identify species of root-knot nematodes: *Meloidogyne arenaria*, *M. hapla*, *M. incognita*, and *M. javanica*. Nematropica 31:273–282.

[R8] Eisenback, J. D., Bernard, E. C., Starr, J. J., Lee, T. A. and Tomaszewski, E. K. 2003. *Meloidogyne haplanaria* n. sp. (Nematoda: Meloidogynidae), a root-knot nematode parasitizing peanut in Texas. Journal of Nematology 35:395–403. 19262770 PMC2620679

[R9] Georgi, L. L. and Abbott, A. G. 1998. Variation in ribosomal genes in *Meloidogyne arenaria*. Fundamental and Applied Nematology 21:685–694.

[R10] Hiatt, E. E. III, Shipe, E. R. and Lewis, S. A. 1988. Soybean response to two isolates of *Meloidogyne arenaria*. Journal of Nematology 20:330–332. 19290218 PMC2618798

[R11] Humphreys-Pereira, D. A., Kim, T. and Park, J. -K. 2021. “Characterization of nematode mitochondrial genomes”, In Perry, R. N., Hunt, D. and Subbotin, S. A. (Eds), Techniques for Work with Plant and Soil Nematodes. CABI, Wallingford, Oxfordhire; Boston, MA pp. 250–264.

[R12] Janssen, T., Karssen, G., Verhaeven, M., Coyne, D. and Bert, W. 2016. Mitochondrial coding genome analysis of tropical root-knot nematodes *(Meloidogyne*) supports haplotype based diagnostics and reveals evidence of recent reticulate evolution. Scientific Reports 6:22591. 26940543 10.1038/srep22591 PMC4778069

[R13] Joseph, S., Mekete, T., Danquah, W. B. and Noling, J. 2016. First report of *Meloidogyne haplanaria* infecting Mi-resistant tomato plants in Florida and its molecular diagnosis based on mitochondrial haplotype. Plant Disease 100:1438–1445. 30686187 10.1094/PDIS-09-15-1113-RE

[R14] Khanal, C., Robbins, R. T., Faske, T. R., Szalanski, A. L., McGawley, E. C. and Overstreet, C. 2016. Identification and haplotype designation of *Meloidogyne* spp. of Arkansas using molecular diagnostics. Nematropica 46:261–270.

[R15] Powers, T. O. and Harris, T. S. 1993. A polymerase chain reaction for the identification of five major *Meloidogyne* species. Journal of Nematology 25:1–6. 19279734 PMC2619349

[R16] Powers, T. O., Mullin, P. G., Harris, T. S., Sutton, L. A. and Higgins, R. S. 2005. Incorporating molecular identification of *Meloidogyne* spp. into a large-scale regional nematode survey. Journal of Nematology 37:226–235. 19262865 PMC2620951

[R17] Subbotin, S. A. 2021a. “Molecular identification of nematodes using polymerase chain reaction (PCR)”, In Perry, R. N., Hunt, D. and Subbotin, S. A. (Eds), Techniques for Work with Plant and Soil Nematodes. CABI, Wallingford, Oxfordhire; Boston, MA, pp. 218–239.

[R18] Subbotin, S. A. 2021b. “Phylogenetic analysis of DNA sequence data”, In Perry, R. N., Hunt, D. and Subbotin, S. A. (Eds), Techniques for Work with Plant and Soil Nematodes. CABI, Wallingford, Oxfordhire; Boston, MA, pp. 265–282.

[R19] Triantaphyllou, A. C. 1981. Oogenesis and the chromosomes of the parthenogenetic root-knot nematode *Meloidogyne incognita*. Journal of Nematology 13:95–104. 19300730 PMC2618073

[R20] Van der Beek, J. G. and Karssen, G. 1997. Interspecific hybridization of meiotic parthenogenetic *Meloidogyne chitwoodi* and *M. fallax*. Phytopathology 87:1061–1066. 18945041 10.1094/PHYTO.1997.87.10.1061

[R21] Ye, W., Robbins, R. T. and Kirkpatrick, T. 2019. Molecular characterization of root-knot nematodes (*Meloidogyne* spp.) from Arkansas, USA. Scientific Report 9:15680. 10.1038/s41598-019-52118-4 PMC6821887 31666613

